# Supplementary material for: Effects of Trichoderma harzianum combined with Phanerochaete chrysosporium on lignin degradation and humification during chicken manure and rice husk composting
Source: Front Microbiol. 2025 Feb 28;16:1515931. doi: 10.3389/fmicb.2025.1515931 (PMC11906335; doi:10.3389/fmicb.2025.1515931)
Supplement: Supplementary file 1 [file Data_Sheet_1.zip › 6.Function/All/FunGuild/l1/PCoA.pdf]

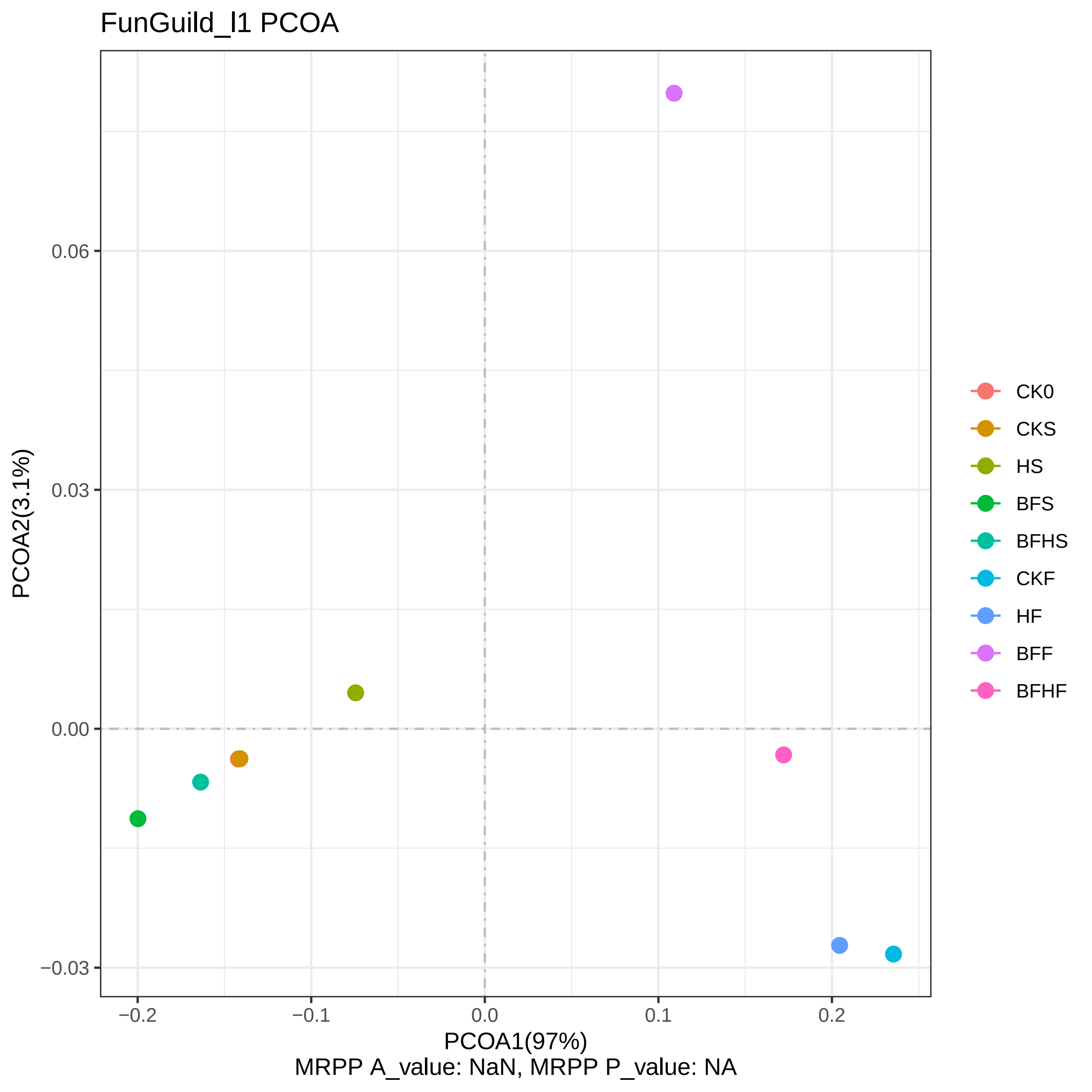
FunGuild_l1 PCOA
0.06
0.03
0.00
−0.03
PCOA2(3.1%)
−0.2
−0.1
0.0
0.1
0.2
PCOA1(97%)
MRPP A_value: NaN, MRPP P_value: NA
CK0
CKS
HS
BFS
BFHS
CKF
HF
BFF
BFHF
